# Copper mediated amyloid-*β* binding to Transthyretin

**DOI:** 10.1038/s41598-018-31808-5

**Published:** 2018-09-13

**Authors:** Lidia Ciccone, Carole Fruchart-Gaillard, Gilles Mourier, Martin Savko, Susanna Nencetti, Elisabetta Orlandini, Denis Servent, Enrico A. Stura, William Shepard

**Affiliations:** 1 CEA Institut des Sciences du Vivant Frédéric Joliot, Service d’Ingènierie Moléculaire des Protéines (SIMOPRO), Université Paris-Saclay, 91191 Gif-sur-Yvette, France; 2 Synchrotron SOLEIL, L’Orme des Merisiers, Saint-Aubin, BP 48, 91192 Gif-sur-Yvette, France; 3 Dipartimento di Farmacia, Universitá di Pisa, Via Bonanno 6, 56126 Pisa, Italy; 4 Dipartimento di Scienze della Terra, Universitá di Pisa, Via Santa Maria 53-55, 56100 Pisa, Italy

## Abstract

Transthyretin (TTR), a homotetrameric protein that transports thyroxine and retinol both in plasma and in cerebrospinal (CSF) fluid provides a natural protective response against Alzheimer’s disease (AD), modulates amyloid-*β* (A*β*) deposition by direct interaction and co-localizes with A*β* in plaques. TTR levels are lower in the CSF of AD patients. Zn^2+^, Mn^2+^ and Fe^2+^ transform TTR into a protease able to cleave A*β*. To explain these activities, monomer dissociation or conformational changes have been suggested. Here, we report that when TTR crystals are exposed to copper or iron salts, the tetramer undergoes a significant conformational change that alters the dimer-dimer interface and rearranges residues implicated in TTR’s ability to neutralize A*β*. We also describe the conformational changes in TTR upon the binding of the various metal ions. Furthermore, using bio-layer interferometry (BLI) with immobilized A*β*(1–28), we observe the binding of TTR only in the presence of copper. Such Cu^2+^-dependent binding suggests a recognition mechanism whereby Cu^2+^ modulates both the TTR conformation, induces a complementary A*β* structure and may participate in the interaction. Cu^2+^-soaked TTR crystals show a conformation different from that induced by Fe^2+^, and intriguingly, TTR crystals grown in presence of A*β*(1–28) show different positions for the copper sites from those grown its absence.

## Introduction

Human transthyretin (TTR), a homotetrameric 127-residue protein, is the main carrier of thyroxine (T4) in celebrospinal fluid (CSF) and the second main carrier in blood^1^. TTR also interacts with other proteins, such as retinol-binding protein (RBP) and thus contributes to retinol transport^2^. In aged patients, TTR can be responsible for certain amyloidotic diseases, when sporadically the tetramer dissociates into conformationally unstable TTR monomers prone to aggregate into TTR-amyloid fibrils^3,4^. Interestingly, TTR also interacts with amyloid-*β* (A*β*) and plays a protective role in Alzheimer’s disease (AD) by sequestering A*β* and reducing proteopathic stress. A*β* is generated upon sequential cleavage of the amyloid precursor protein (APP), and an imbalance between A*β* production and brain clearance has been postulated as a possible cause of A*β*-amyloid deposition in AD^5^. TTR binds soluble, oligomeric and A*β* fibrils^6,7^ playing a role in A*β* clearance^8^. The precise mechanism by which TTR binds to A*β* remains unknown and is particularly difficult to pinpoint because of the plethora of aggregated forms of A*β*.

Metals ions such as Zn^2+^, Mn^2+^, Cu^1+,2+^, Fe^2+,3+^ affect A*β* fibril formation and toxicity inducing a profusion of different conformations^9–13^. The same cations also interact with TTR. High concentrations of Zn^2+^ and Cu^2+^ (but not the iron cations) can promote the formation of a TTR-amyloid complex *in vitro*, while chelators (EDTA or EGTA) can disrupt fibrils composed of aggregated TTR^14^. Zinc ions boost L55P-TTR fibril formation^15^ and promote TTR metallopeptidase activity^16^. Such activity is also induced by other ions, such as Co^2+^, Mn^2+^ and Fe^2+^, but not Cu^2+^ or Ni^2+^ ^17^.

Currently, AD drugs are mainly supportive or palliative. All potentially curative drugs have failed during or before phase III trials^18^. Recently, trials of two anti-A*β* monoclonal antibodies, bapineuzumab and solanezumab, for AD patients with mild-to-moderate symptoms, were stopped due to a lack of beneficial clinical outcomes^19^. Clearly, the strategy of inhibiting A*β* is too simplistic. It would be beneficial to comprehend how A*β* is cleared from the brain naturally, and what mechanisms are employed by TTR to scavenge A*β*. The structure of human TTR is well known^20–22^, and hypothetical models for its interaction with A*β* have been proposed. A recent NMR study places A*β*(12–28) within an external pocket spanning across the epigallocatechin-3-gallate (EGCG) binding site^23^. Previous studies positioned A*β* in the TTR interior pocket extending towards the short *α*-helix^7^, involving L110 and L82, since their mutation (L → A) destroys TTR’s ability to bind A*β*^24^. L110 is located in the central hydrophobic channel, and for A*β* to bind in proximity, a rearrangement of the TTR monomers would be required, as the available volume is insufficiently small in the channel of the standard TTR tetramer^25^.

Senile amyloid plaques contain high levels of Cu, Fe and Zn^26^ that can promote polymorphic A*β* aggregation^13,27^. We question whether metal-aggregated forms might interact better with TTR, and whether the TTR-A*β* interaction might be modulated by metal ions. Although the zinc binding sites of TTR have already been elucidated in the cryptic peptidase form of TTR^28,29^, the positioning of A*β* within the TTR proteolytic site remains to be determined. Apart from zinc, the binding of other metal ions to TTR has not yet been characterized crystallographically.

**Figure 1:**
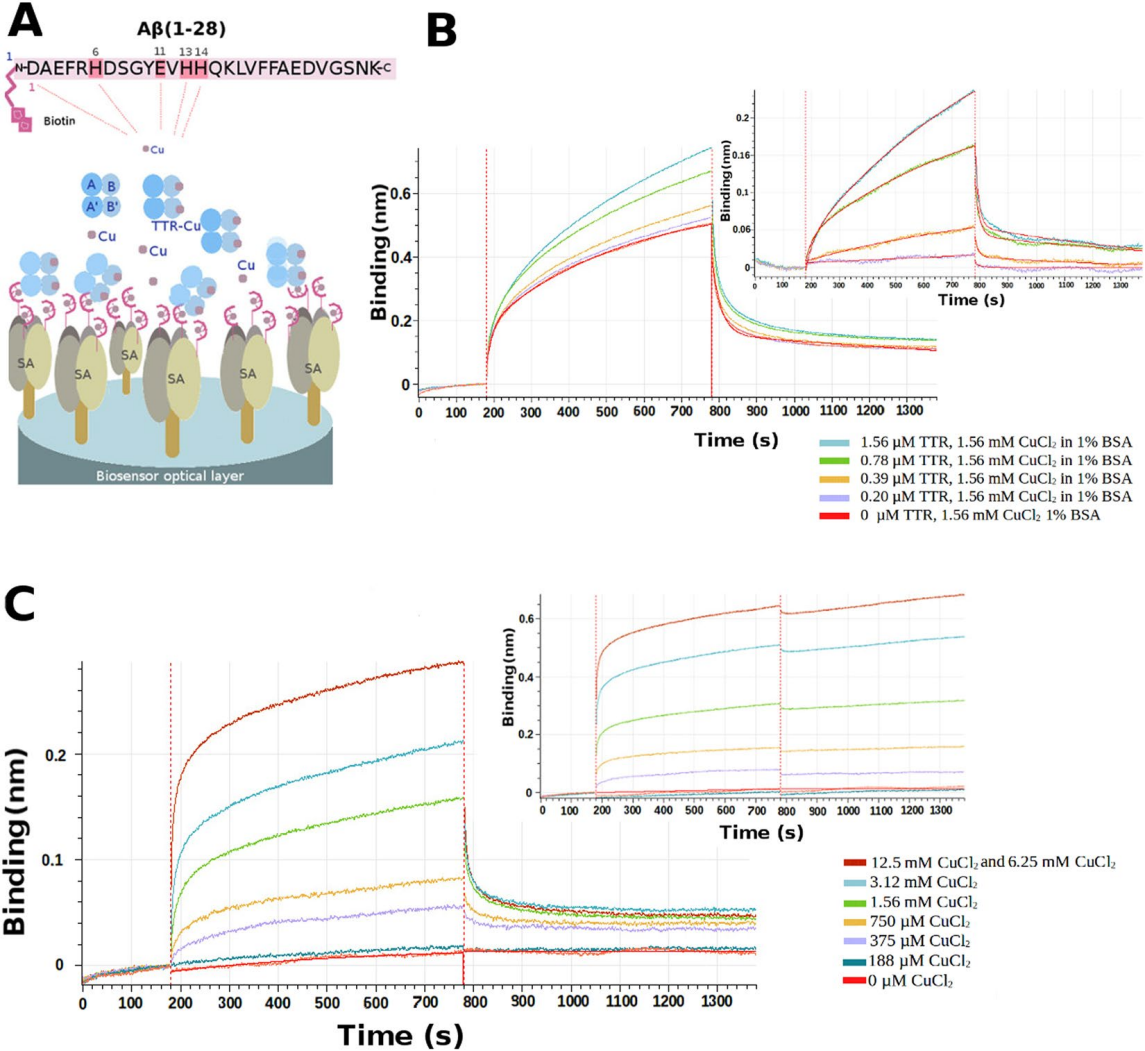
Copper-mediated TTR-A*β* interactions. (**A**) Overall scenario for bio-layer interferometry (BLI) using a streptavidin (SA) coated optical sensor. Biotinylated A*β*(1–28) was loaded on the BLI bio-sensor tip surface (100 nM). A single biotin is present at the N-terminal position of the A*β* peptide. The tip was then plunged in solutions containing TTR and copper at different concentrations to analyze the interactions. (**B**) Sensorgram curves colored according to TTR concentration (0 *μ*M–1.56 *μ*M). Inset on the right, represents the specific time dependent binding of TTR on immobilized A*β*(1–28) in the presence of 1% of BSA at pH 5.5, corresponding to the total binding for each TTR concentration minus the nonspecific binding found without TTR in presence of 1.56 mM A*β* and 1% of BSA. (**C**) Time dependent binding of TTR on immobilized A*β* peptide at a concentration of 1.56 *μ*M of TTR in presence of various CuCl_2_ concentrations at pH 5.5 with 1% of BSA without CuCl_2_ during the dissociation. The sensorgram curves are colored according to CuCl_2_ concentration (0 mM–12.5 mM). Inset on the right, shows the dissociation step using buffer added by various CuCl_2_ concentrations (0 to 12.5 mM).

**Figure 2:**
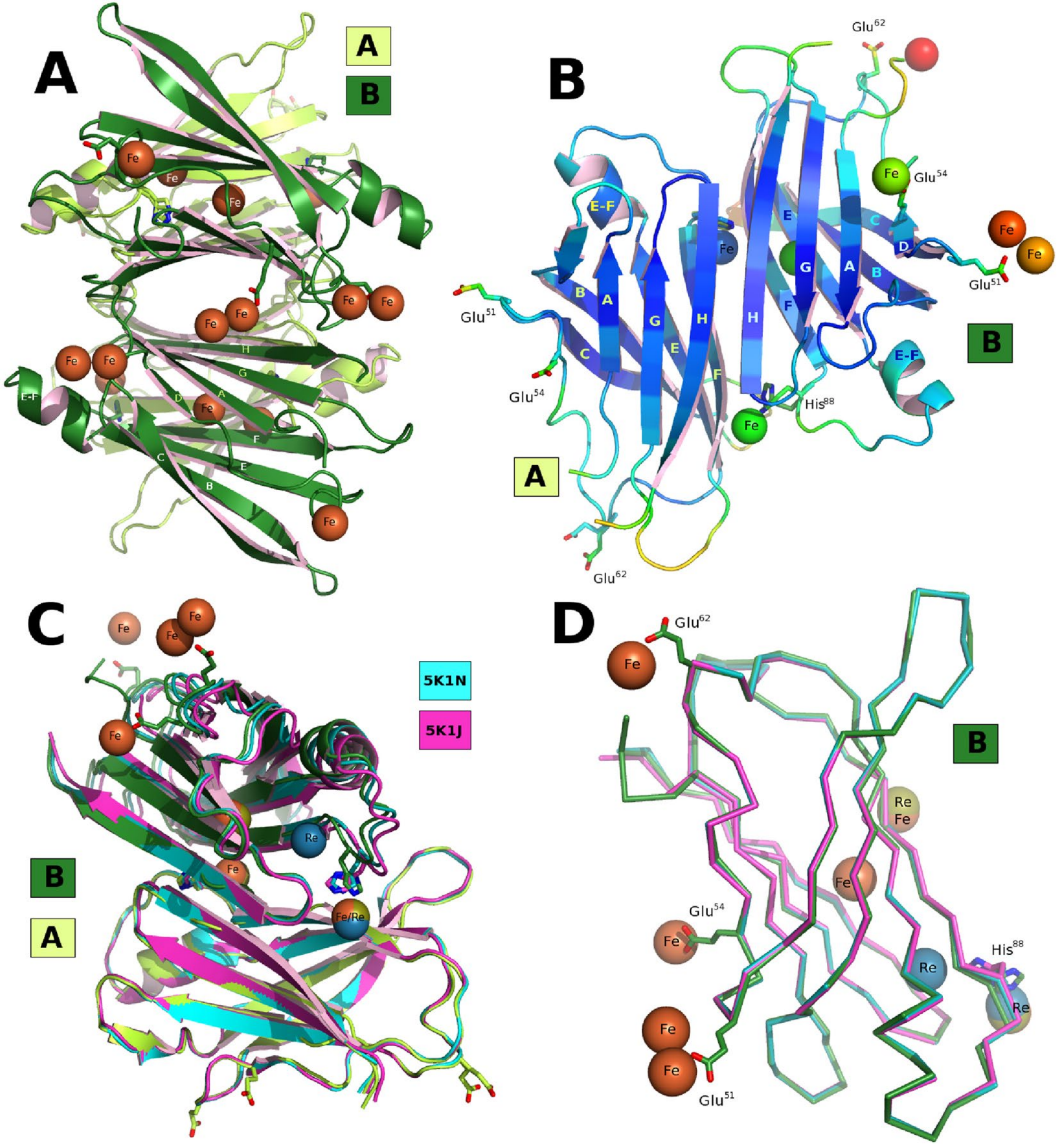
Conformation of the TTR-Fe complex and comparison with the TTR-Re complex. (**A**) Overall view of the tetrameter highlighting the iron positions with monomer *A* and *B* in light and dark green, respectively. (**B**) Overall view of the hetero-conformational *A*-*B* dimer colored according to the crystallographic B-value. (**C**) Superposition of the dimers of the Fe *vs*. Re TTR complexes showing that additional changes occur in the *B* monomer increasing its difference from that of the *A* monomer. (**D**) Superimposition of monomers *B* from the two rhenium complexes against the TTR-Re complex^30^.

Here we report the interaction between TTR and A*β* in the presence of Cu^2+^ obtained using bio-layer interferometry (BLI) with a biotinylated peptide comprising the 1–28 region of A*β* (Fig. 1). We also present the crystal structures of Fe^2+^, Mn^2+^ and of Cu^2+^ bound to TTR (Table 1) crystallized in the presence and absence of A*β*(1–28). Soaking TTR crystals with CuCl_2_ or FeCl_2_ at acidic pH induces a change in conformation comparable to that observed for the TTR-rhenium complex^30^ (Fig. 2). We hypothesize that the conformational change induced by Cu and Fe is related to TTR’s ability to bind A*β*.

**Table 1:** Crystallization conditions and data collection statistics.

Structure	TTR-Fe	TTR-Mn	TTR-Cu	TTR-Cu-A*β*
**PDB code**	**5N5Q**	**5N62**	**5N7C**	Not deposited
Metal-Ligand	Fe	Mn	Cu	Cu-A*β*
Crystallization	27% polyethylene glycol 4,000 (PEG4K), 0.2 M imidazole malate, pH 6.0	26% PEG4K, 0.2 M imidazole malate, pH 6.0	21% PEG4K, 0.14 M imidazole malate, pH 6.0, 3.6% MPEG 5 K, 0.03 M sodium acetate, pH 5.5	Co-crystallization with A*β* 1–28 and CuCl_2_ in 21% PEG4K, 0.14 M imidazole malate, pH 6.0, 3.6% MPEG, 5 K, 0.1 M sodium acetate, pH 5.5
Cryoprotectant	40% SM3*, 25% MPEG 5 K, 0.1 M CHC (90% acid/10% basic), 30 mM FeCl_2_, 2 h soak.	40% CM7*, 25% MPEG 5 K, 5 mM MnCl_2_, 10 min soak.	40% SM3*, 25% MPEG 5 K, 0.1 M CHC(90% acid/10% basic), 30 mM CuCl_2_ 30 min soak.	40% CM1*, 25% MPEG 5 K, 0.1 M CHC (90% acid/10% basic), 30 mM CuCl_2_ 30 min soak.
**Data Collection**	**anomalous**	**anomalous**	**anomalous**	**non anomalous**
Source	Soleil Proxima-2	Soleil Proxima-2	Soleil Proxima-2	Soleil Proxima-2
Wavelength (Å)	1.739153	1.891993	1.175919	0.980035
Space group	P2_1_2_1_2	P2_1_2_1_2	P2_1_2_1_2	P2_1_2_1_2
Unit-cell (Å)	42.82 83.10 65.34	43.27 85.83 63.90	42.99 82.54 67.77	43.10 82.75 67.14
Molec./asym.	2	2	2	2
Resolution (Å)	50–2.53/2.68–2.53	50–1.8/1.85–1.80	50–2.45/2.59–2.45	50–2.14/2.27–2.14
CC_1/2_ (%)	99.6/30.0	99.8/39.0	99.8/89.0	99.9/52.5
〈*I*/*σ*(*I*)〉	8.28/0.83	11.53/1.52	14.75/3.29	13.0/1.10
R-merge (%)	20.0/198	17.6/217	10.7/67.2	12.9/215
R-factor (%)	18.5/182	16.3/202	9.9/62.1	12.4/222
Completeness (%)	99.2/95.3	100/100	99.5/96.7	99.8/98.9
Multiplicity	13.21/6.87 (anomalous)	7.37 (anomalous)	13.7/7.0 (anomalous)	13.1
**Refinement**	**REFMAC5**	**Phenix**	**REFMAC5**	**REFMAC5**
Resolution (Å)	40.91–2.53/2.60–2.53	38.63–1.80/1.89–1.80	41.06–2.45/2.51–2.45	38.23–2.43/2.49–2.43
No. of reflections	8113/548 (non-anomalous)	22723/2658 (anomalous)	8868/467 (non-anomalous)	9042/650 (non-anomalous)
R-work	19.2/41.0	19.5/28.4	16.9/20.3	20.9/31.0
R-free	25.3/40.4	24.4/33.2	25.4/43.1	29.7/44.4
RMSD Bond lengths (Å)	0.013	0.009	0.009	0.015
RMSD Bond angles (°)	1.6	1.025	1.714	1.773
Ramachandran favored	96.0%	98.0%	94.0%	96.9%
Ramachandran outliers	0	0	0	3

Cryoprotection: SM1: 12.5% diethylene glycol + 12.5% glycerol + 12.5% 1,2-propanediol + 25% DMSO + 25% 1,4-dioxane; SM3: 25% diethylene glycol + 25% ethylene glycol + 25% glycerol + 25% 1,4-dioxane; CM1: 12.5% diethylene glycol + 37.5% 1,2-propanediol + 12.5% DMSO; CM7: 12.5% di-ethylene glycol + 12.5% ethylene glycol + 12.5% glycerol + 25% 1,2-propanediol + 12.5% DMSO^68^. Cryoprotectant solution is formulated with 40% v/v mixed compounds (CM), 50% v/v precipitant and 10% v/v buffer^69^. CHC: Linear mixed buffer composed of citric acid, HEPES and CHES; acid mix at pH 4.0, basic at pH 10.0^70^. CC_1/2_: Data quality correlation coefficient^71^. Data collection statistics are from XDS^60^. Refinement statistics are from REFMAC5^63^ or phenix.refine (Phenix)^65^.

## Results

### Binding affinity of Transthyretin with wt-A*β* peptide 1–28

Biotin-tagged peptide of the wild type-A*β* fragment 1–28 (wt-A*β*) was immobilized on a streptavidin-coated BLI tip (Octet Red 96 platform, Pall FortèBio) (Fig. 1A). Several solutions of 1.56 mM of CuCl_2_ in 50 mM acetate buffer, pH 5.5, 1% BSA with variable amounts (0, 0.20, 0.39, 0.78, and 1.56 *μ*M) of TTR were tested and the binding saturation was observed at 1.56 *μ*M. The apparent K_D_ of wt-A*β*, calculated in presence of 1.56 mM of CuCl_2_ was estimated to be 54.70 ± 1.19 nM (Fig. 1B). In order to evaluate the influence of Cu^2+^ in the kinetic parameters of this interaction, various concentrations of CuCl_2_ were tested during the association step (Fig. 1C). In addition, a second experiment has been done to investigate the effect of Cu^+2^ in the dissociation step (Fig. 1C, insert). Different solutions of 1.56 *μ*M TTR in 50 mM acetate buffer, pH 5.5, 1% BSA with 0, 188, 375, 750 *μ*M and 1.56, 3.12, 6.25 and 12.5 mM of CuCl_2_ were tested. No significant binding was observed with CuCl_2_ below 188 *μ*M. Starting at 375 *μ*M, the signal progressively increased reaching a maximum at 6.25 mM and 12.5 mM which represents the saturation signal (Fig. 1C). When the Cu^2+^ is added in the dissociation step, the trimeric complex remains stable and no dissociation was observed (Fig. 1C).

The same experiment carried out with ZnCl_2_ instead of CuCl_2_ showed no binding. When the experiment is performed at physiological pH (pH 7.4) in presence of CuCl_2_ the K_D_ is close to the value found at pH 5.5, while with ZnCl_2_ no association was observed. For iron, it was not possible perform the same experiments due to the iron-induced denaturation of TTR and its subsequent precipitation (see Supplementary Fig. S1).

Complementary experiments were carried out with two control peptides, one with a scrambled sequence (sm-A*β*) and another with the three histidines and a glutamate^31^ mutated to alanine (mut-A*β*). In presence of 1.56 mM of CuCl_2_ with 1.56 *μ*M of TTR, the mutated peptide was found to bind with a K_*D*_ = 685 ± 68 nM that is ten times less than the wt-peptide. No binding was observed for the scrambled peptide.

### Identifying specific metal binding sites and key inter-residue distances

Human TTR crystals were soaked directly in metal-containing cryoprotectant solutions. The duration, pH and metal salt concentration were optimized to allow any conformational changes to occur with minimal deterioration to the X-ray diffraction quality. In order to unambiguously identify metal sites, X-ray diffraction data were collected at two X-ray wavelengths just prior and just beyond the X-ray absorption K-edge of the corresponding metal (see Supplementary Information). The phased anomalous difference Fourier maps were used as a guide to identify and position the specific metal ions (see Supplementary Fig. S2).

Three key inter-residue distances can be used to describe the conformational changes induced by metal binding to TTR. Differences in the *A*-*A*′ and *B*-*B*′ Leu-110–Leu-110 separation (see Supplementary Table S3) measures the asymmetric rotation of the dimers relative to each other. The Asp-38–Asp-38 separation between the *A* and *B* monomers is a monitor of the change in shape of the dimer, while the Thr-123*A*–Gly-83*B* distance changes as residues 72–92 on monomer *B* shifts relative to *A*. This distance varies in response to the conformational change that displaces His-88.

### Iron binding to TTR

Human TTR crystals are particularly sensitive to FeCl_2_ soaking experiments. Once optimized, the crystal packing was found to be affected by the TTR conformational changes, and the unit cell parameters alter from *a* = 43.3 Å, *b* = 85.8 Å, *c* = 63.9 Å, as observed for the TTR-Mn complex, and typical for most human TTR-ligand complexes in the PDB^32^, to *a* = 42.8 Å, *b* = 83.1 Å, *c* = 65.3 Å, for the TTR-Fe complex (Table 1, PDB code: 5N5Q).

The TTR-Fe structure with the strongest anomalous signal in the phased anomalous difference Fourier map, out of 35 successful soak experiments, is reported here (see Supplementary Table S1). The observed conformational changes surpass those induced by rhenium^30^. The magnitude of these changes, combined with an increased total absorbed dose at the X-ray absorption K-edge of Fe^33^ and the higher multiplicity to improve the anomalous signal^34^, results in a structure with a crystallographic resolution limit lower than that for TTR-Re.

The largest anomalous features for the TTR-Fe complex are located in proximity of Glu-51. On *A* monomers, the electron density can be fitted by a three-atom Fe-cluster (see Supplementary Fig. S2B) and on *B* monomers by two Fe atoms (Fig. 2C). An additional Fe site at the entrance of the T4 binding site involves Glu-54 from symmetry related *B* and *B*′ monomers belonging to the same tetramer.

Some uninterpreted electron density extends beyond the phased anomalous difference density for the Fe, suggesting the presence of a chelating residue, probably Glu-7 from the disordered N-terminus. An additional unidentified peak, too strong be explained by a water molecule, but without any anomalous signal and thus can not be an Fe atom, is located close to His-88 (see Supplementary Fig. S2D). The TTR tetramer loses the symmetry of the T4-TTR complex (PDB code: 1ICT^22^) matching the asymmetric conformation of the TTR-Re-complex^30^. In this conformation, the spatial relationship between monomers *A* and *B* is altered giving the dimer a different shape. The conformational change involves only the *B* monomer and affects the *β*-strands E and F and the short E-F *α*-helix. In total, sixteen Fe atoms (including the clusters in interaction with Glu-51) are bound to each tetramer chelated by just three residues from each monomer (see Supplementary Tables S1 and S2).

The conformation of the TTR-Fe complex, with an Asp-38–Asp-38 separation of 25.7 Å is intermediate compared to the two TTR-Re complexes^30^. The large shift in the stretch of residues 72–92 of *B* relative to *A* with a Thr-123*A*–Gly-83*B* distance of 27.0 Å and a large Leu-110–Leu110 asymmetry attests to the largest conformational changes observed in TTR structures to date.

### Manganese binding to TTR

Although manganese is of minor interest for either AD^35^ or TTR amyloidogenesis because the Mn concentrations are 1000-fold lower than either Cu or Fe in the CSF^36^, its position in the periodic table relative to Fe and Re warranted the determination of its complex. When TTR crystals grown at pH 6.0 were soaked in 5 mM MnCl_2_ under conditions similar to those carried out with Fe, Mn was found bound to Glu-66 and Asn-98 in monomer *B* and to Asp-99 and Glu-66 in monomer *A*, with minor local changes. The residues involved in Mn binding are not involved in Fe or Re binding (see Supplementary Fig. S3, PDB code: 5N62). More globally, Mn may influence an *A*-*A*′ and *B*-*B*′ asymmetry. The separation between the central Leu-110 residues (*B*-*B*′ Leu-110–Leu110 separation of only 6.73 Å) suggests a rotation of the TTR dimers in a direction opposite to that observed for Fe and Re-TTR structures (see Supplementary Table S3). The conformation of the TTR-Mn complex with a distance Asp38–Asp38 of 19.2 Å is within the range for distances observed for wt-TTR with ligands (e.g. 5EZP^37^) and even a wide selection of mutant TTR structures including those subjected to heating (PDB code: 2QEL^38^) or to a pH as low as 4.6 (PDB code: 2G4G^39^).

### Copper binding to TTR

When TTR crystals grown or soaked in 30 mM CuCl_2_ the conformation of the TTR-Cu complex yields a distance Asp-38–Asp-38 of 25.0 Å, a Thr-123–Gly-83 distance of 25.7 Å and a Leu-110–Leu-110 asymmetry, which are comparable in magnitude but opposite to that of the TTR-Mn complex. This suggests a more nuanced response to metal binding (see Supplementary Table S2, PDB code: 5N7C).

**Figure 3:**
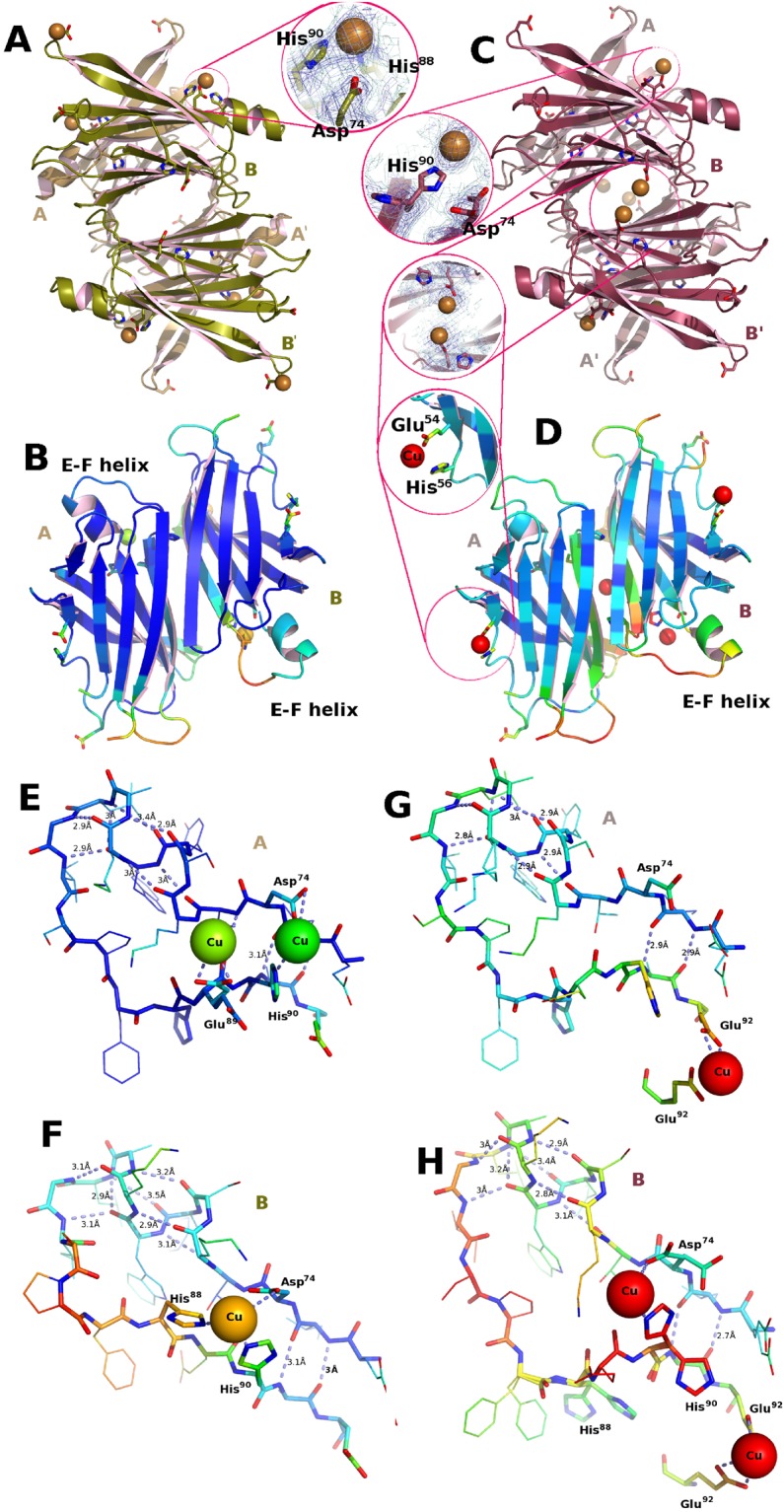
Conformation induced by copper binding to TTR. (**A**,**B**) Structure obtained in the absence of A*β*. (**C**,**D**) Structure obtained using crystals grown in the presence of A*β*(1–28). Copper soaking into TTR crystals stimulates in both cases a change in conformation similar to that observed for iron and for rhenium. The amplitude of the changes are greater in TTR crystals grown in the absence of A*β*. (**A**) Overall view of the hetero-conformational *A*-*B*/*A*′-*B*′ tetramer rebuilt using the 2-fold crystallographic symmetry operation. (**B**) Crystallographic *A*-*B* dimer colored according to B-value to highlight the increased mobility that occurs in the 74–92 stretch that includes the E-F helix. (**C**) In the crystals obtained in the presence of A*β*(1–28) the positioning of the copper is different from those grown in the absence of the amyloid peptide. The binding is probably weakened from copper chelation by disordered A*β*(1–28) in the solvent channels of the crystal. Residual copper binding is observed in proximity of Asp-74 with minor cooperation of His-90 and at the entrance of the ligand binding channel mediated by Glu-92 with His-56 in proximity. (**D**) The overall mobility of the protein structure for the TTR crystals grown in the presence of A*β*(1–28) is greater than for those grown without the peptide. (**E**) In the crystals obtained in the presence of A*β*(1–28) the positioning of the copper is different from those grown in the absence of the amyloid peptide. Residual copper binding is observed in proximity of Asp-74 with minor cooperation of His-90 and at the entrance of the ligand binding channel mediated by Glu-92 with His-56 in proximity. (**F**) The overall mobility of the structure for the crystals grown in the presence of A*β*(1–28) is greater than for those grown without the peptide. (**G**) Copper binds only at the entrance of the ligand binding channel on monomer A. (**H**) The disorder of stretch of residues 72–92 of monomer B is probably due to an incomplete transition towards the conformation observed in (**D**).

The overall structure of the complex is illustrated in Fig. 3. The main difference that distinguishes this complex and others is in the positioning of the copper ion chelated between His-88, His-90 and Asp-72 on monomer B (Fig. 3D) and the binding between His-90 and Asp-72 on monomer *A* (Fig. 3D) which endows the stretch of residues 72–92 with a conformation different from those of the other TTR-metal complexes.

### Copper binding to TTR crystals grown in presence of A*β*

Contrary to other metal ions, Cu does not transform TTR into a protease that can cleave A*β*^17^, and this permits their co-crystallization without the possibility of A*β* proteolytic degradation. Crystallographic analysis of the crystals obtained from solutions of TTR and A*β* do not show any convincing electron density corresponding to the A*β* peptide, although some uninterpretable density is apparent nearby. Similarly, crystals grown in the presence of A*β* and CuCl_2_ also failed to incorporate the peptide in an ordered manner. However, it is noteworthy that when soaked with CuCl_2_ (conditions as for the TTR-Cu complex PDB code: 5N7C; *this work*), the crystals grown in the presence of A*β* show a reduced binding of copper and an incomplete transformation, with B-values higher than those for other complexes (PDB code: not deposited, see Fig. 3F).

Based on the magnitude of the three key distances, the extent of the conformational changes is intermediate between the TTR-Mn and the TTR-Cu complexes (see Supplementary Table S3). The electron density for the 72–92 zone in proximity of His-90 suggests the coexistence of modified and unmodified *B* monomers. The conformation of the unmodified monomers matches that of the A monomers, while the *B* monomers adopt a conformation different from that found in the TTR-Cu complex (Fig. 3 compare zoom A *vs*. zoom E and D *vs*. H). Note that His-88 does not participate in metal chelation and a Cu^2+^ ion is chelated by Glu-92 (Fig. 3G,H) and Glu-54 (Fig. 3E,F) at the *A*–*B* the *B*–*B*′ interfaces, respectively. The latter TTR-metal interaction is common to the TTR-Fe complex (see Supplementary Tables S1 and 2; Fig. 2).

## Discussion

TTR functions as a transporter in plasma and in the brain of several molecules, such as T4 and retinol. In the CSF, TTR is also recognized as the main A*β* binder, and, it is able to transport A*β* across the brain-blood barrier in the brain-to-blood direction^8^. The details of this clearance mechanism, however, remain to be elucidated.

The concentration of TTR is different in the blood and in the CSF: 3–4 *μ*M and 0.1–0.4 *μ*M, respectively^24^. Under physiological conditions, the soluble A*β* peptide is secreted into the synaptic cleft of normal subjects and AD patients, and its concentration is in the nanomolar range^40^. In the brain, the concentration of A*β* is about six-times higher than in plasma. Perturbations in the efflux of A*β* out of the brain could affect the levels of soluble A*β* in the CSF^12,41^. Moreover, when metals ions are released into the synaptic space during neurotransmission, they can modify the stability of the A*β* peptide. In the AD affected brain, the total concentration of Cu and other metals contained in amyloid plaque deposits has been established at 400 *μ*M^26^.

In this work, we have investigated the interaction between TTR and A*β* by using a segment (residues 1–28) of the full length A*β* peptide as it is more soluble and stable in buffer solutions. More importantly, however, A*β*(1–28) contains the hydrophobic core VFF (residues 18–20), which is recognized by TTR^7,23^. Furthermore, A*β*(1–28) holds the residues that chelate metal ions^31^ and that can mediate the interaction between TTR and A*β*. It is not known if TTR binds the entire sequence of A*β* or only the fragments of A*β* cleaved by TTR^6,42^, but the segment 1–28 of A*β* is exposed in amyloid fibrils of A*β*(1–42)^43^.

Our BLI study has revealed that the affinity of TTR for A*β*(1–28) is modulated by copper. The binding of TTR to immobilized A*β*(1–28) increases with CuCl_2_ concentration from 0 to 12.5 mM (Fig. 1). When CuCl_2_ is included in the BLI dissociation buffer, no dissociation is observed Fig. 1C, consistent with the hypothesis that Cu^2+^ plays a key role in the stability of the TTR-Cu-A*β* complex.

Under pathological conditions, the pH dramatically decreases in the CNS, in plasma and in cells^44–46^. It is known that copper binding to histidines enhances A*β* aggregation at the pH typical of physiological acidosis^47^. The pH dependence of this interaction supports the involvement of the histidine side-chains. Consequently, the majority of our BLI and crystallographic experiments were done at acid pH (pH 5.5). Indeed, acidic pH and elevated CuCl_2_ concentration in the crystal soaking experiments (Table 1) favors a change in the TTR conformation in the stretch of residues 72–92 (see Supplementary Fig. S3).

The metal ion concentration needed to induce the conformational change in TTR crystals is necessarily elevated compared to the conditions in solution as the system is constrained by lattice packing forces. When TTR crystals of the *P*2_1_2_1_2 crystal form are soaked in FeCl_2_ at acidic pH, only the *B* monomer in the asymmetric unit changes its conformation, whereby the E-F helix and the stretch of residues 85–92 undergo a rearrangement that is accompanied by a variation in the dimer-dimer interface. A similar change in conformation was observed when *P*2_1_2_1_2 TTR crystals were soaked with a Re-complex^30^. Although the binding of various metals provokes similar changes, the changes induced differ in their extent. Three key distances can be used to evaluate the magnitude of the conformational change (see Supplementary Table S3): the separation between the four Ile-110 residues in the tetramer (to monitor the rotation of the dimers relative to each other), the distance between Gly-83*B* on the E-F loop from Thr-123*A* (to measure the shift of the E-F helix), and the separation between two Asp-38 residues (to assess the relationship between the monomers that form the dimer). This last measure provides a distinctive signature of metal binding. The Asp-38–Asp-38 separation, typically 21.5 Å in the absence of metals, shortens to 20.3 Å in the TTR-Mn complex and to 17.8 Å for the TTR-Zn complex (PDB code: 3DGD^28^), while in the TTR-Cu complex it increases to 25.7 Å and 26.3 Å in the TTR-Re complex (PDB code: 5K1N^30^) up to a maximum of 27.0 Å for the TTR-Fe complex (see Supplementary Table S3).

Metal chelation by proteins often involves the repositioning of certain residues. When considering TTR-A*β* complexes it is important to take into account that both partners in the interaction may alter their conformation in a dynamic and polymorphic manner. Fe promotes A*β* aggregation^48^ that interferes with the dynamics of amyloid formation^49^, and its metal binding is pH-dependent and affects oligomerization^50^. Morphological differences may affect toxicity. A*β* aggregates containing Zn, Fe or Cu are neurotoxic^51,52^.

Studies *in vitro* are focused on A*β* interactions with Cu and Zn, even if Mn also binds A*β* peptides^31^ and plays a role in neurodegeneration. In order to investigate the importance of Mn in the TTR-A*β* interactions, TTR crystals were soaked in 5 mM of MnCl_2_ at pH 6.0. No significant conformational changes were observed, suggesting that even if Mn binds A*β*^31^, it does not play a relevant role in the interactions between TTR and A*β*.

Although Cu^2+^ induces a conformational change in TTR (Fig. 3; X-ray anomalous differences validate the presence Cu sites see Supplementary Table S2) which resembles those revealed by the Fe^2+^ and Re^2+^ experiments, the manner in which Cu^2+^ is chelated differs from those observed for Fe^2+^ or Re (Fig. 2). Even if both His-88 and His-90 are involved in Cu and Zn chelation, the interaction from the third residue is different, being Asp-74 for Cu^2+^ (Fig. 3) and Glu-92 in the case of Zn^2+^ at pH 4.6^28^. The network of residues that intervene in metal binding is quite extensive (see Supplementary Fig. S3), and most residues have been recognized as possible zinc binders. The mutation of His-90 to Ala results in the loss of TTR catalytic activity and its ability to disrupt fibrils^42^.

Despite the fact that the TTR-A*β* interaction can be demonstrated in the presence of Cu by BLI, TTR crystals grown in the presence of CuCl_2_ and A*β* did not show any ordered A*β* peptides. This implies that the crystallization forces of TTR molecules disrupt the TTR-A*β* complex. Given the size of the A*β*(1–28) peptide with respect to TTR monomers (127 aa), one would expect the TTR-A*β* complex to crystallize in rather different unit cell constants and/or space group.

However, the TTR crystal structure contains 30Å -wide diamond-shaped solvent channels, which run the length of the crystal, and the likely presence of A*β* in the crystal interstices could be inferred from lower Cu^2+^ binding due to A*β* chelation. Under identical soak conditions, the TTR conformational change is characterized by a smaller Asp-38–Asp-38 separation (23.9 Å) compared to that seen in the absence of A*β* (25.0 Å). An additional Cu binding site is located in proximity of Glu-54 (Fig. 3E,F). This residue has been identified in regards to Cr^3+^ binding, and its involvement in Cu^2+^ binding has been suggested^53^. Thus, Glu-54 could act as the gatekeeper to the tunnel that leads to the four Leu-110 residues at the center of the tetramer, a residue that when mutated to alanine abolishes TTR’s A*β* scavenging activity. A contact between the A*β* peptide and Leu-110 would require a transit in front of Glu-54.

Our results show that TTR changes its conformation in response to binding Fe^2+^ ^54^ (as opposed to Fe^3+^ as confirmed by XANES experiments, see Supplementary Information Fig. S4) and Cu^2+^ at acidic pH, and that Cu is essential for the recognition of A*β*(1–28) by TTR. Only certain divalent metals ions (Cu^2+^, Fe^2+^) provoke the crystallographic conformational change. When TTR is soaked with trivalent metal ions under acid pH conditions (Al^3+^, Gd^3+^, or Fe^3+^), no conformational changes were observed.

The ability of Cu to promote the formation of the TTR-A*β* complex, and presumably the cerebral clearance of A*β*, is consistent with the report that mice with a defective Cu transporter (which removes Cu) have higher Cu levels, a reduced number of amyloid plaques and diminished plasma A*β*^55^. The lack of binding A*β* by TTR in the absence of Cu, may appear to be in contradiction with experiments carried out with 125I-labeled A*β* (1–40) and A*β*(1–28) peptides by Schwarzman *et al*., when it was shown that TTR recognizes A*β* in CSF fluid, inhibits its aggregation and prevents fibril formation^5^. However, excluding the possibility that the iodinated peptide used might interact *via* any of the three TTR halogen binding pockets in the TTR central tunnel, the results may also imply that in the absence of a metal, different polymorphic forms of A*β* are recognized by TTR. By stabilizing a A*β* conformation with mild affinity for TTR, Cu^2+^ ions rescue the interaction. TTR may also bind with higher affinity polymeric A*β* which acquires an alternative and stable conformation through A*β*-A*β* interactions. The immobilization method used here involves A*β*(1–28) mono-biotinylated at the N-terminus (Fig. 1B), and the results are consistent with studies showing that non-aggregating A*β* fragments do not quench TTR tryptophan fluorescence^56^. It is possible that the same peptide, if allowed to dimerize *via* the LVFFA stretch, may also adopt a conformation that is recognized by TTR in the absence of Cu. The polymorphic diversity of the longer A*β* (1–40/1–42) peptides, with extensive hydrophobic stretches, is likely to generate stable conformations with higher affinities for TTR. Modifying the BLI to function with more complex aggregates may be challenging, but will be necessary to guide the crystallographic structure determination of the TTR-A*β* complex with and without metals.

To conclude, the TTR-Cu conformation provides an alternative starting point for the design of molecules that aim to stabilize a form of TTR with an enhanced A*β*-scavenging activity to counter the reduction of TTR expression in AD patients^57^. As such, these results emphasize Cu as a “ forgotten factor” upon which the TTR-A*β* interaction may depend. Furthermore, metal chelation therapies, which are useful to reduce oxidative stress, should also take into account the essential levels of Cu required for A*β* clearance.

## Methods

### Synthesis of biotinylated A*β* peptides

Fmoc-amino acids, Fmoc-pseudoproline dipeptide, and 2-(6-chloro-1H-benzotriazole-1-yl)-1,1,3,3,-tetra-methylaminium hexafluorophosphate (HCTU) were obtained from Novabiochem (Darmstadt, Germany). N-biotine-NH-(PEG)_11_-COOH and biotinamidohexanoic acid N-hydroxysuccinimide ester were purchased at Merck and Sigma Aldrich respectively. The resin and all the peptide synthesis grade reagents (N-methylpyrrolidone (NMP), N-methylmorpholine (NMM), dichloromethane, piperidine, trifluoroacetic acid (TFA), anisole, thioanisole, and triisopropylsilane) were purchased from Sigma (Saint-Quentin Fallavier, France). The synthesis of peptide A*β*(1–28) wild type (DAEFRHDSGYEVHHQKLVFFAEDVGSNK), scrambled A*β* peptide (1–28) (LNHQEKGDRFSDYEVAFSVGHKVDFHEA) and mutant (DAEFRADSGYAVAAQKLVFFAEDVGSNK) were performed on a Protein Technologies, Inc., Prelude Synthesizer at a 25 *μ*mol scale using a 10-fold excess of Fmoc-amino acid relative to the preloaded Fmoc-Lys(Boc)-wang-LLresin (0.33 *μ*mol/g) or Fmoc-Ala-wang-LLresin (0.33 *μ*mol/g). Fmoc-protected amino acids were used with the following side chain protections: tert-butyl ester (Glu and Asp), tert-butyl ether (Ser and Tyr), trityl (His, Asn, and Gln), tertbutoxycarbonyl (Lys), and 2,2,5,7,8-pentamethyl-chromane-6-sulfonyl (Arg). Amino acids were coupled twice for 5 min using 1:1:2 amino acid/HCTU/NMM in NMP. In scrambled A*β* peptide (1–28) a pseudoproline dipeptide (FS) was used at positions 10–11 and 17–18 and coupled twice for 10 min. After incorporation of each residue, the resin was acetylated for 5 min using a 50-fold excess of a mixture of acetic anhydride and NMM in NMP. Fmoc deprotection was performed twice for 3 min using 20% piperidine in NMP, and 30 sec NMP top washes were performed between deprotection and coupling and after acetylation steps. Biotinylation of A*β* peptides (1–28) was done with 50 mg resin after Fmoc deprotection of the N-terminal residue, using a 10-fold excess of either N-biotin-NH-(PEG)_11_-COOH (A*β* peptide 1–28 wild type and mutant) and HCTU and NMM as coupling reagents (see above) or biotinamidohexanoic acid N-hydroxysuccinimide ester in NMP (scrambled A*β* peptide). After completion, the peptidyl-resins were treated with a mixture of TFA/thioanisole/anisole/TPS/water (82:5:5:2.5:5) for 2 h. The crude peptides were obtained after precipitation and washes in cold ethyl ether followed by dissolution in 10% acetic acid and lyophilization. The different peptides were purified by reverse phase HPLC using an X-Bridge BHE C_18_-300-5 semi-preparative column (Waters, USA) (250 × 4.6 mm; 4 mL × min^−1^; solvent A: H_2_O/TFA 0.1%; solvent B: acetonitrile/TFA 0.1% using a gradient of 0–60% solvent B into A in 60 min). The purity of each peptide was checked by mass spectrometry using ESI-MS (Bruker,Germany). Biotinylated A*β* peptide (1–28) wild type calculated m/z: 4088.5, found: 4088.0; biotinylated mutant A*β* peptide (1–28) calculated m/z: 3832.2, found m/z: 3832.1 and biotinylated scrambled A*β* peptide (1–28) calculated m/z: 3601.5, found m/z: 3601.8.

### Binding affinity measurements by bio-layer interferometry

Binding between TTR and A*β* peptides (wild type, scrambled and mutant) in absence or presence of copper was performed in real time by bio-layer interferometry using an Octet Red 96 platform instrument (Pall FortéBio Corp., Menlo Park, CA). Streptavidin bio-sensors were hydrated in 50 mM acetate, pH 5.5, 1% BSA (buffer A) for over 20 minutes before the start of each run and then loaded with 100 nM of biotinylated A*β*(1–28). A single run was subdivided into five distinct steps as follows^58^: (i) Baseline determination, in which the streptavidin bio-sensor tip was immersed in buffer A for 180 s to set the baseline; (ii) Loading step, during which 100 nM A*β* was immobilized on to the streptavidin-coated bio-sensor tip for 80 s; (iii) Back-soak step, during which the bio-sensor tip was again immersed in buffer A for 180 s to remove excess unbound A*β*; (iv) Association step, during which the A*β* loaded bio-sensor tip was immersed in TTR solutions for 600 s; (v) Dissociation step, during which the tip with TTR-A*β* bound was immersed in the same buffer for 600 s to dissociate the TTR. The data from these two last steps were used to estimate differences in interference caused by binding of TTR to the A*β* loaded tip. A blank run was carried out with buffer A for calibration purposes. A series of 5 runs was performed with CuCl_2_ at constant 1.56 mM, the first without TTR followed by 4 runs with increasing amounts of TTR (0, 0.20, 0.39, 0.78, 1.56 *μ*M. Another series of 8 runs was performed with TTR at constant 1.56 *μ*M, the first without CuCl_2_ followed by 7 runs with increasing amounts of CuCl_2_ (0, 188, 375, 750, 1.56 *μ*M and 1.56, 3.12, 6.25, 12.5 mM. Affinity (K_D_) was calculated using the FortéBio Data Analysis HT v10.0.1.7 software.

### Crystal preparation and structure determination

TTR crystals were grown by sitting drop vapor diffusion as previously reported^59^ using lyophilized human TTR (Calbiochem, Merck Millipore, Darmstadt, Germany) dissolved, 1 mg in 100 mL of 0.02% (w/v) NaN_3_ and dialyzed overnight against 0.1 M NaCl, 50 mM sodium acetate, pH 5.5. The reservoir solution consisted of 21% polyethylene glycol 4,000 (PEG4K), 0.14 M imidazole malate, pH 6.0 and the second one from 21% polyethylene glycol 4,000 (PEG4K), 0.14 M imidazole malate, pH 6.0, 3.6% polyethylene glycol monomethylether (MPEG5K), 30 mM sodium acetate, pH 5.5. Streak seeding was used to induce nucleation. The cryoprotectant solution was composed of 40% of SM2 (12.5% ethylene glycol, 12.5% glycerol, 12.5% 1,2-propanediol, 25% DMSO and 37.5% 1,4-dioxane) 25% PEG 8K and 30 mM of CuCl_2_, MnCl_2_ or FeCl_2_, to which 2.5% H_2_O_2_ or 0.8 mM Methylene Blue (MB) was added in some experiments. The combination of H_2_O_2_ and MB resulted in the loss of the blue color of MB. Crystals were flash cooled by in liquid nitrogen and data were collected on beamline Proxima-2A at the Soleil storage ring in Saint Aubin, France, on a Dectris Eiger 9M detector. Data processing was carried out at the synchrotron facility using XDS^60^ with the *xdsme* script^61^. The structure was solved by molecular replacement using Phaser^62^ followed by refinement using REFMAC5^63^ starting with a TTR model without inhibitor. The electron density maps were viewed and fitted in COOT^64^. The structures were subjected to over ten cycles of rebuilding and refinement with REFMAC5^63^ and PHENIX^65^. The phased anomalous difference Fourier maps were calculated with the program ANODE^66^. Data processing and refinement statistics are given in Table 1. The coordinates for the TTR wild-type and various mutants, see Supplementary Table S3, were retrieved from the Protein Data Bank^32^. The figures were made with PyMOL^67^. Structures deposited into the PDB Data Bank: 5N5Q, 5N62 and 5N7C.

## Electronic supplementary material

Supplementary material
