# Supplementary material for: Copper mediated amyloid-β binding to Transthyretin
Source: Sci Rep. 2018 Sep 13;8:13744. doi: 10.1038/s41598-018-31808-5 (PMC6137083; doi:10.1038/s41598-018-31808-5)
Supplement: Supplementary file 1 — Supplementary material [file 41598_2018_31808_MOESM1_ESM.pdf]

Copper mediated amyloid-β binding to Transthyretin

Lidia Ciccone[1,2], Carole Fruchart-Gaillard[1], Gilles Mourier[1], Martin Savko[2], Susanna Nencetti[3], Elisabetta Orlandini[4], Denis Servent[1], Enrico A. Stura[1], and William Shepard[2,*]

[1]CEA Institut des Sciences du Vivant Frédéric Joliot, Service d′Ingénierie Moléculaire des Protéines (SIMOPRO), Université Paris-Saclay, 91191 Gif-sur-Yvette, France
[2]Synchrotron SOLEIL, L′Orme des Merisiers, Saint-Aubin, BP 48, 91192 Gif-sur-Yvette, France
[3]Dipartimento di Farmacia, Universitá di Pisa, Via Bonanno 6, 56126 Pisa, Italy
[4]Dipartimento di Scienze della Terra, Universitá di Pisa, Via Santa Maria 53-55, 56100 Pisa, Italy
[*]william.shepard@synchrotron-soleil.fr

Influence of metals in amyloid-beta binding to Transthyretin

In order to study the oligomerization state of TTR (Fig. S1) in the presence or absence of Aβ 1-28, under the influence of different metals, namely Cu, Zn and Fe, this complex was run on a native gel by electrophoresis. No change in molecular weight was observed, suggesting that Aβ in the presence of Cu or Zn does not affect TTR oligomerization. However, Fe modifies the TTR migration profile indicating aggregation.

Phased Anomalous Difference Fourier maps

In order to confirm the location of metal ions in the TTR structures obtained with copper and iron soaked crystals, high multiplicity X-ray diffraction data sets at X-ray energies below and above the absorption K-edges for that metal were collected. In these experiments the anomalous signal is low at energies corresponding to those below (pre-) the absorption K-edge (f” < 0.5 electrons), but much stronger at energies corresponding to those above (post-) absorption K-edge (f” 3.8 electrons). Phased anomalous difference Fourier (DANO) maps were calculated using refined models of TTR, and then the DANO peak heights were compared for pre- and post-edge X-ray data sets.

Table S1. Three possible TTR-iron binding sites

Fe Site 5N5Q*	Residues	DANO Height 7.1 keV
1	Glu51 (A)	6.2
	Glu51 (B)	6.0
2	Glu54-His56 (A)	3.1
	Glu54-His56 (B)	4.1
3	His88 (A)	no site
	His88 (B)	3.2

*This work; The table shows the peak r.m.s.d. values present in the phase anomalous difference (DANO) Fourier map corresponding to the three possible iron binding sites. The data were collected at the iron energy 7.1 keV (1.7463 Å)

Table S2. TTR Copper and Iron possible binding sites

Cu Site	Residues	DANO Height		Fe Site	Residues	DANO Height	
		8.9 keV pre-edge	9.0 keV post-edge			7.0 keV pre-edge	7.2 keV post-edge
1	Glu54-His56 (A)	2.1	5.1	1	Glu54-His56 (A)	1.5	3.9
	Glu54-His56 (B)	< 2	4.3		Glu54-His56 (B)	3.1	4.7
2	His90-Glu92 (A)	2.4	4.6	2	Glu51 (A)	2.9	4.2
	His90-Glu92 (B)	< 2	4.8		Glu51 (B)	2.0	2.4
				3	His88 (A)	no site	
					His88 (B)	3.6	3.1

This work; Fe K-edge 7.112 keV, f" = 0.47 e at 7.1 keV; f" = 3.86 e at 7.2 keV Cu K-edge 8.9789 keV f" = 0.49 e at 8.9 keV; f" = 3.88 e at 9.0 keV.

Tables S1, S2 show the results obtained from the phased anomalous difference Fourier maps which identify the positions of the metals ions, despite the relatively moderate diffraction limits of the crystals. In each case, the X-ray diffraction data were collected at both pre-edge and post-edge energies from the same crystal. Different and well-separated positions were chosen on the crystals for the pre- and post-edge collections so that with the use of a micro-focused beam the radiation damage effects could be reduced. From previous experiments with such maps, fully occupied sites for anomalously scattering atoms are typically characterized by peak heights of 6 r.m.s.d. or above. This is not the case for the Fe (PDB_id: 5N5Q Table S1) and Cu TTR structures. Thus the results obtained from these phased anomalous difference Fourier maps should be interpreted as metal sites that are partially occupied or disordered, but unambiguously present (Table S2).

Comparison of metal positions

The residues found to chelate Fe and Cu are given in Tables S1 and S2. The positions of the metal ion sites are different in the case of iron compared to copper (Fig. S2E). Regarding copper binding, the Cu sites are different for crystals grown in the presence of Aβ and soaked with $CuCl_2$ compared to those that were grown in the absence of Aβ and soaked using the same experimental protocol (Fig. S2B). The residues involved in metal chelation in crystals grown in the presence of Aβ change their conformation with respect with crystals grown without Aβ. His-90*A* coordinates a Cu atom together with Asp-74*A* without Aβ, but in the presence of Aβ it is transferred to Glu-92*A* and Glu-92*B*, nearby (Fig. S2B). The presence of the Aβ peptide either in the interstitial spaces or bound in many alternative conformations has a strong influence on the TTR conformation of monomer B, affecting mainly the conformational heterogeneity (Fig. S2C). The comparison of the binding of Fe and Mn to TTR is illustrated in Fig. S3 showing the involvement of Asn-98 and Glu-99 in monomer *A* and of Asp-86 in monomer *B* in Mn binding. In order to chelate the metal, the protein must undergo a small local conformational change (Fig. S3B).

XANES experiments to determine the iron oxidation state in a Fe-soaked TTR crystal

To determine the oxidation state of the Fe bound to TTR in its crystal structure, we have measured X-ray absorption near edge structure (XANES)[1] at Fe K-edge. Two stock solutions of Fe(II) and Fe(III) at 100 mM were prepared by dissolving $FeCl_2$ and $Fe(NO_3)_3$ salts in H_2O, respectively. The solutions were left at 20°C overnight. The TTR crystal was soaked for 2h in a cryo-solution containing a final concentration of 30 mM $FeCl_2$ before freezing. The fluorescence spectra of both iron solutions (II and III) and of the TTR crystal were recorded by scanning the incident X-ray energy across the K-absorption edge of iron (7.112 keV). The Fe K-edge XANES spectrum of the Fe-soaked TTR crystal matches the spectrum for $FeCl_2$, whereas the $Fe(NO_3)_3$ spectrum is displaced to higher energies by about 5 eV, which is typical for Fe(III) complexes. The comparison of the XANES spectra provides additional evidence that only Fe(II) is bound to TTR (Fig. S4).

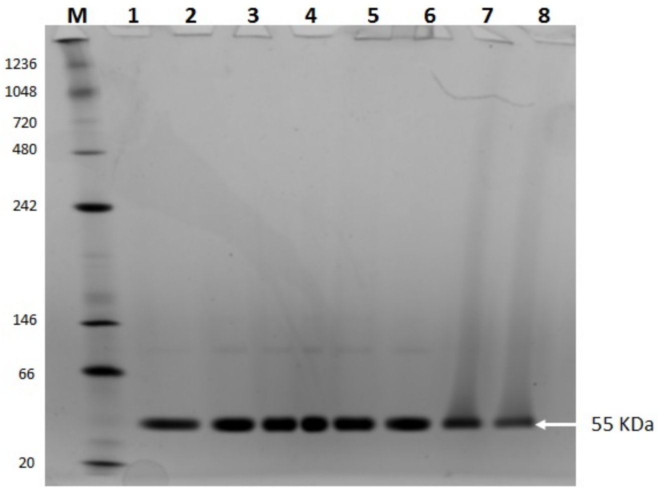

Figure S1. X-ray fluorec, Aβ 1-28 and in the trimeric complex TTR-Cu-Aβ(1-28). (*A*) Study of the oligomerization state of TTR with or without Aβ(1-28) peptide and with or without different metals. 1-D native electrophoresis is used to determine native mass and oligomeric state of proteins. In lane 1, the band at 55 kDa corresponds to the TTR tetramer. In absence (lanes 1, 3, 5 and 7) or presence of Aβ(1-28) peptide (lanes 2, 4, 6 and 8) or $CuCl_2$ (lanes 3 and 4) or $ZnCl_2$ (lanes 5 and 6), neither change was observed (lane 2 to 6). On the other hand, in the presence of $FeCl_2$ (lanes 7 and 8) the migration profile is different and suggests a degradation of the TTR.

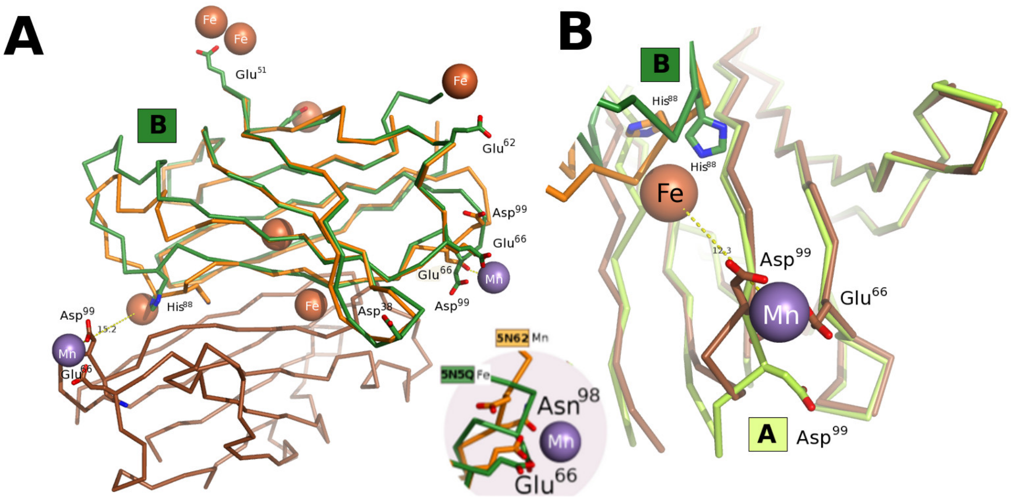

Figure S3. Comparison of the binding of Fe and Mn to transthyretin. (*A*) Fe and Mn TTR complexes aligned on monomers B. The proximity of the Mn binding site to His-88 on the other monomer is to be noted. The iron-manganese separation is 15.2 Å. (*B*) Fe and Mn TTR complexes aligned on monomers A. Mn is chelated by Glu-66 and Asp-99 giving a shorter iron–manganese separation of 12.3 Å. The conformation of the loop carrying Asp-99 on monomer A differs from that of monomer B and from that of the Fe-TTR complex. This is significant as the monomer A conformation of the Fe and Mn TTR complexes are otherwise identical. The comparison of the metal positions in the two structures suggests a possible mechanism for the transfer of a metal ion from a storage site on Glu-66 to the inaccessible His-88 that in the absence of a conformational change cannot reliably acquire a metal ion.

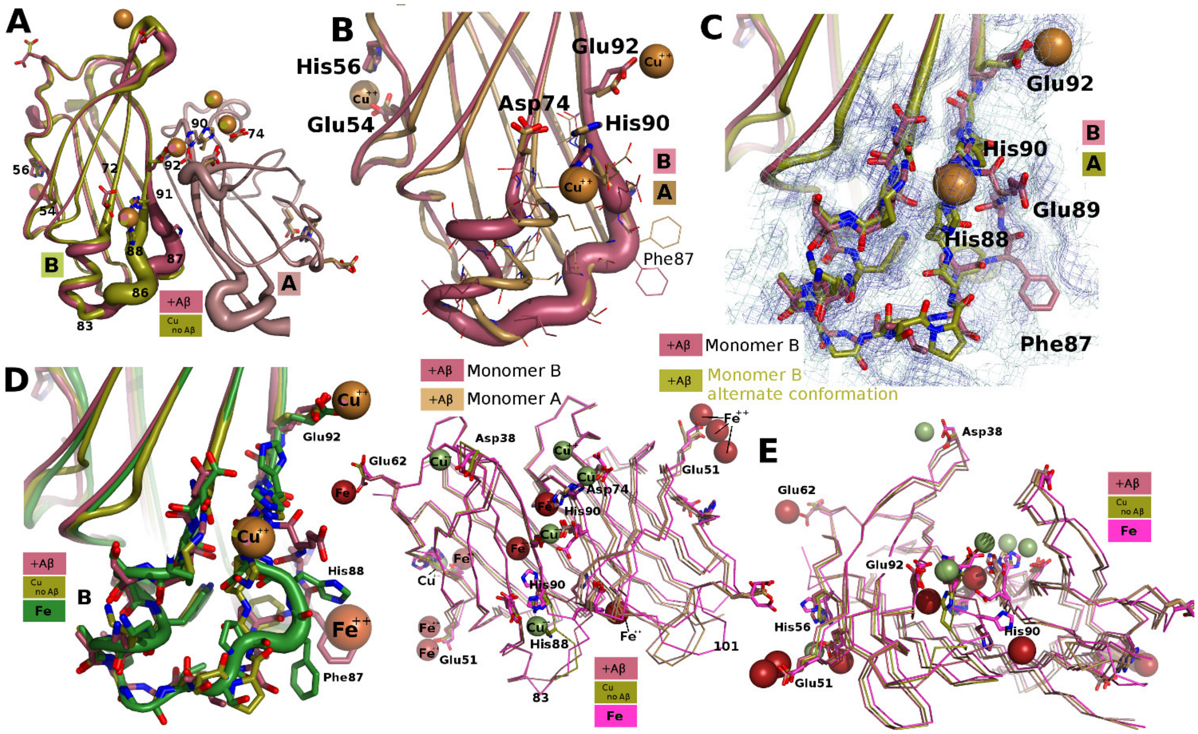

Figure S2. Comparison of copper and iron binding to transthyretin. (*A*) Superimposition of the structures obtained as a result of crystal soaking in $CuCl_2$ in "putty" cartoon representation showing the most flexible regions of the protein in thicker tube diameters. The crystals grown in the presence of Aβ(1-28) (copper brown color) show a larger structural deviation compared to those grown without (copper green color). (*B*) Superimposition of monomers *A* (ochre) and *B* (copper brown) to show the conformational difference of the two monomers in crystals grown in the presence of Aβ(1-28) soaked in $CuCl_2$. (*C*) The electron density in the 72-92 residue region of the $CuCl_2$ soaked Aβ grown crystals is ambiguous and might represent a superimposition of multiple conformations, dominated by that shown in panel B with some *B* molecules maintaining the more canonical *A* conformation. (*D*) Superimposition of the 72-92 residue region from the structures obtained as a result of the crystal soaking in $CuCl_2$ (grown with and without Aβ) and $FeCl_2$ showing the full spectrum of conformational variations. (*E*) Two perpendicular views of the metal sites for the three structures shown in panel D displaying the variability in the metal positions.

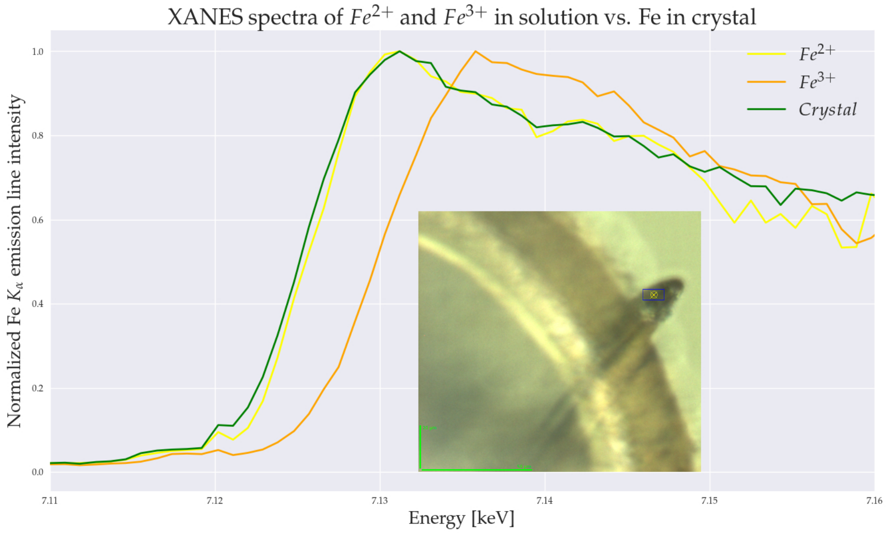

Figure S4. XANES of Fe^{2+} and Fe^{3+} solutions *versus* spectrum measured on a Fe-soaked TTR crystal. In yellow the XANES of the solution of Fe^{2+}, in orange the spectrum of the solution of Fe^{3+}, and in green the spectrum measured on the TTR crystal soaked with Fe^{2+}. The insertion shows the exact position of the crystal where the XANES spectrum was measured, the blue box shows the size of the beam.

Table S3. Separation between TTR residues

PDB code	mutation<br>metal	distance (Å)<br>Asp-38–Asp-38	distance (Å)<br>Thr-123–Gly-83
5EZP[2]	WT[4]	A-B 17.1	A-B 21.3
		C-D 19.2	C-D 21.7
		E-F 19.1	E-F 21.5
		G-H 18.2	G-H 21.6
2G4G[3]	WT[a]	A-B 18.7	A-B 21.8
3D7P[4]	WT[a]	A-B 20.1	A-B **25.3**
3CBR[4]	WT[a]	A-B **25.2**	-
**5N62***	TTR-Mn[a]	A-B 19.2	A-B 20.3
**5N5Q***	TTR-Fe[a]	A-B **25.7**	A-B **27.0**
**5N7C***	TTR-Cu[a]	A-B **25.0**	A-B **25.7**
	TTR-Cu-Aβ[a]	A-B **23.9**	A-B **24.2**
5K1J[5]	TTR-Re[8]	A-B **23.9**	A-B **25.4**
5K1N[5]	TTR-Re[12]	A-B **24.7**	A-B **26.3**
3DGD[6]	F87M/L110M	A-B ***13.2***	A-B **17.8**
	Zn^{2+}	C-D ***13.4***	C-D **17.8**
4TNF[n]	K15W	A-B 18.9	A-B 21.5
1ETB[7]	V30M	A-B 19.2	A-B 21.2
2QEL[8]	G53S/E54D/L55S[h]	A-B 18.4	A-B 20.4
		C-D 16.3	C-D 21.8
1G1O[9]	G53S/E54D/L55S	A-B 19.0	A-B 22.5
		C-D 16.8	C-D 20.7
4TKW[10]	L55P	A-B 19.9	A-B 21.8
5TTR[11]	L55P	A-B 20.8	A-B 21.0
		C-D 19.8	C-D 20.9
		E-F 21.1	E-F 21.2
		G-H 20.5	G-H 20.7
3DO4[n]	T60A[a]	A-B 19.1	A-B 21.2
		C-E 19.4	C-E 21.5
		D-F 20.8	D-F 21.2
		G-H 20.5	G-H 21.3
2G3Z[3]	I84A[a]	A-B 18.2	A-B **17.5**
2G3X[3]	I84S[a]	A-B 18.1	A-B **12.7**
4I87[12]	I84S[a,c]	A-B 18.1	A-B 22.2
4TL5[10]	S85P	A-B 18.7	A-B 21.4
1SOQ[13]	A108Y/L110E	A-A'21.0	A-A' 21.5
		B-B'18.7	B-B' 19.7
		C-D 18.1	C-D 21.1
1F86[11]	T119M	A-B 18.9	A-B 21.8
4TNG[n]	T119M	A-B 18.5	A-B 21.1
4HIS[14]	V122I[t]	A-B 19.8	A-B 20.9

PDB code	mutation<br>metal	distance (Å)<br>Leu-110–Leu-110	distance (Å)<br>Leu-110–Leu-110
5EZP[2]	WT[4]	A-C 7.83	B-D 7.94
		E-H 7.85	F-D 7.80
**5N62***	TTR-Mn[a]	A-A' 7.47	B-B' 6.73
**5N5Q***	TTR-Fe[a]	A-A' **5.81**	B-B' **8.54**
**5N7C***	TTR-Cu[a]	A-A' 6.98	B-B' 7.46
	TTR-Cu-Aβ[a]	A-A' 7.03	B-B' 7.50
5K1J[5]	TTR-Re[8]	A-A' 7.24	B-B' 7.88
5K1N[5]	TTR-Re[12]	A-A' **6.47**	B-B' **8.55**
3DO4[n]	T60A[a]	A-C 6.86	B-E 7.31
		D-H 7.79	F-G 6.94
1G1O[9]	G53S/E54D/L55S	A-D 7.08	B-C 7.33
2G3Z[3]	I84A[a]	A-A' 6.90	B-B' **10.42**
2NOY[3]	I84S	A-A' 6.99	B-B' 6.62

This work; [a]Acidic pH: 2G4G at pH 4.6, 3D7P at pH 4.0, 3CBR at pH 3.5 - Thr-123A–Gly-83B cannot be evaluated as 76B-85B is disordered in the structure ; [4]TTR-wt with 4-hydroxy-chalcone; [h]heated TTR; [t]TTR in complex with Tafamidis; [c]TTR in complex with CHF5074, [n]PDB entries without an associated publication.

References

1. Evans, G. & Pettifer, R. F. Chooch: a program for deriving anomalous-scattering factors from x-ray fluorescence spectra. *J. applied crystallography* **34**, 82–86 (2001).
2. Polsinelli, I. *et al.* A new crystal form of human transthyretin obtained with a curcumin derived ligand. *J. structural biology* **194**, 8–17 (2016). DOI 10.1016/j.jsb.2016.01.007.
3. Pasquato, N. *et al.* Acidic ph-induced conformational changes in amyloidogenic mutant transthyretin. *J. Mol. Biol.* **366**, 711–719 (2007).
4. Palaninathan, S. K., Mohamedmohaideen, N. N., Snee, W. C., Kelly, J. W. & Sacchettini, J. C. Structural insight into pH-induced conformational changes within the native human transthyretin tetramer. *J. molecular biology* **382**, 1157–67 (2008). DOI 10.1016/j.jmb.2008.07.029.
5. Ciccone, L., Policar, C., Stura, E. A. & Shepard, W. Human TTR conformation altered by rhenium tris-carbonyl derivatives. *J. structural biology* **195**, 353–364 (2016).
6. Palmieri, L. d. C. *et al.* Novel Zn2+-binding sites in human transthyretin: implications for amyloidogenesis and retinol-binding protein recognition. *The J. biological chemistry* **285**, 31731–41 (2010). DOI 10.1074/jbc.M110.157206.
7. Hamilton, J. A. *et al.* The x-ray crystal structure refinements of normal human transthyretin and the amyloidogenic Val-30–>Met variant to 1.7-Å resolution. *The J. biological chemistry* **268**, 2416–24 (1993).
8. Karlsson, A. & Sauer-Eriksson, A. E. Heating of proteins as a means of improving crystallization: A successful case study on a highly amyloidogenic triple mutant of human transthyretin. *Acta Crystallogr. Sect. F: Struct. Biol. Cryst. Commun.* **63**, 695–700 (2007).
9. Eneqvist, T., Andersson, K., Olofsson, A., Lundgren, E. & Sauer-Eriksson, A. E. The beta-slip: a novel concept in transthyretin amyloidosis. *Mol. cell* **6**, 1207–18 (2000). DOI 10.1016/S1097-2765(00)00117-9.
10. Saelices, L. *et al.* Uncovering the mechanism of aggregation of human transthyretin. *J. Biol. Chem.* **290**, 28932–28943 (2015). DOI 10.1074/jbc.M115.659912.
11. Sebastião, M. P., Lamzin, V., Saraiva, M. J. & Damas, A. M. Transthyretin stability as a key factor in amyloidogenesis: X-ray analysis at atomic resolution. *J. Mol. Biol.* **306**, 733–744 (2001).
12. Zanotti, G. *et al.* Structural evidence for native state stabilization of a conformationally labile amyloidogenic transthyretin variant by fibrillogenesis inhibitors. *FEBS Lett.* **587**, 2325–2331 (2013).
13. Hörnberg, A., Olofsson, A., Eneqvist, T., Lundgren, E. & Sauer-Eriksson, A. E. The beta-strand D of transthyretin trapped in two discrete conformations. *Biochimica et biophysica acta* **1700**, 93–104 (2004). DOI 10.1016/j.bbapap.2004.04.004.
14. Penchala, S. C. *et al.* AG10 inhibits amyloidogenesis and cellular toxicity of the familial amyloid cardiomyopathy-associated V122I transthyretin. *Proc. Natl. Acad. Sci. United States Am.* **110**, 9992–7 (2013).
